# Dehydroepiandrosterone Sulfate (DHEAS) Stimulates the First Step in the Biosynthesis of Steroid Hormones

**DOI:** 10.1371/journal.pone.0089727

**Published:** 2014-02-21

**Authors:** Jens Neunzig, Rita Bernhardt

**Affiliations:** Department of Biochemistry, Faculty of Technical and Natural Sciences III, Saarland University, Saarbrücken, Germany; Clermont Université, France

## Abstract

Dehydroepiandrosterone sulfate (DHEAS) is the most abundant circulating steroid in human, with the highest concentrations between age 20 and 30, but displaying a significant decrease with age. Many beneficial functions are ascribed to DHEAS. Nevertheless, long-term studies are very scarce concerning the intake of DHEAS over several years, and molecular investigations on DHEAS action are missing so far. In this study, the role of DHEAS on the first and rate-limiting step of steroid hormone biosynthesis was analyzed in a reconstituted *in vitro* system, consisting of purified CYP11A1, adrenodoxin and adrenodoxin reductase. DHEAS enhances the conversion of cholesterol by 26%. Detailed analyses of the mechanism of DHEAS action revealed increased binding affinity of cholesterol to CYP11A1 and enforced interaction with the electron transfer partner, adrenodoxin. Difference spectroscopy showed *K*_d_-values of 40±2.7 µM and 24.8±0.5 µM for CYP11A1 and cholesterol without and with addition of DHEAS, respectively. To determine the *K*_d_-value for CYP11A1 and adrenodoxin, surface plasmon resonance measurements were performed, demonstrating a *K*_d_-value of 3.0±0.35 nM (with cholesterol) and of 2.4±0.05 nM when cholesterol and DHEAS were added. Kinetic experiments showed a lower K_m_ and a higher k_cat_ value for CYP11A1 in the presence of DHEAS leading to an increase of the catalytic efficiency by 75%. These findings indicate that DHEAS affects steroid hormone biosynthesis on a molecular level resulting in an increased formation of pregnenolone.

## Introduction

**Figure 1:**
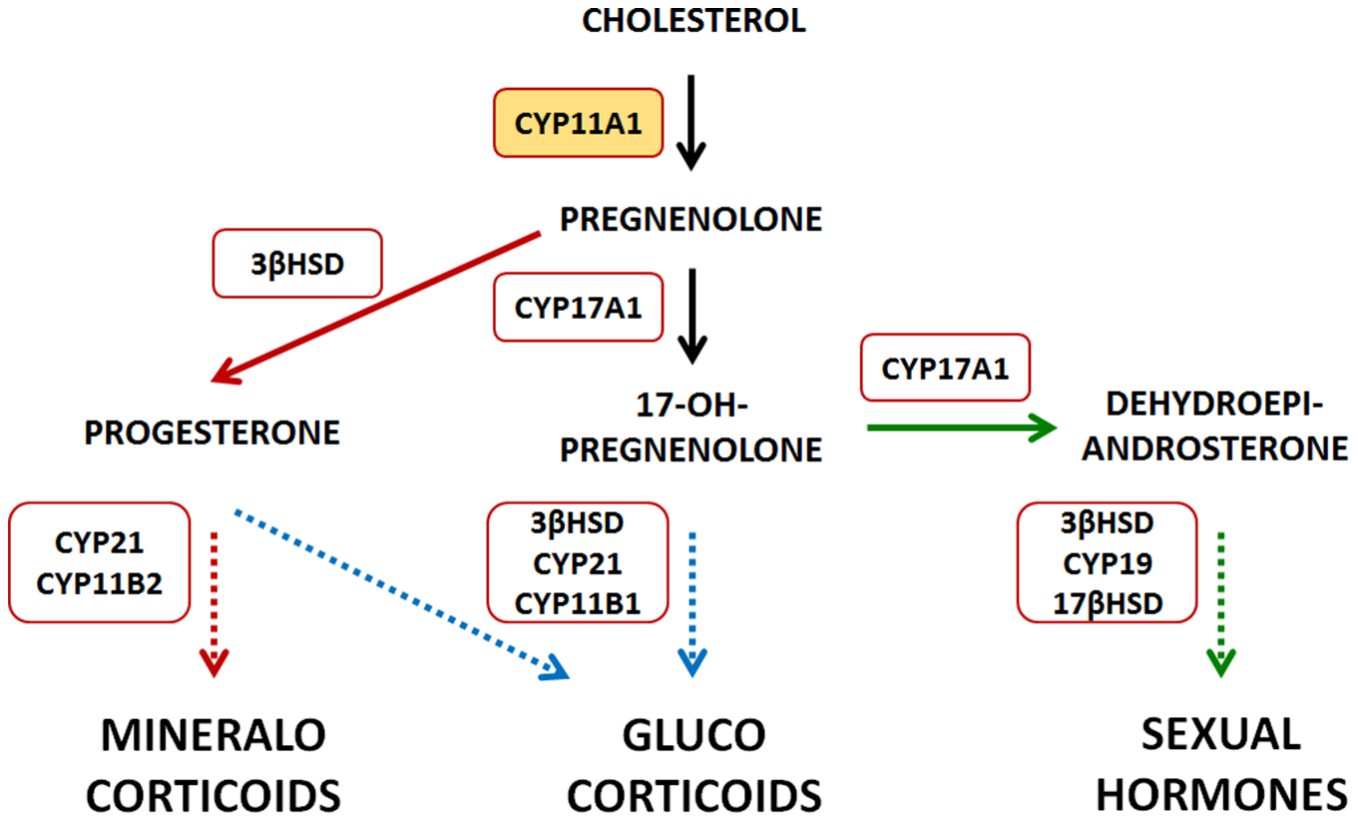
Schematic overview of the steroid hormone biosynthesis.

In mammalian organisms, steroid hormones are indispensable for a normal development. Considering their action, these steroids are classified into three main groups. Mineralocorticoids, with aldosterone as the most important representative, regulate the salt household and hence the blood pressure. Cortisol belongs to the glucocorticoids and provides the organism with energy stimulating the gluconeogenesis. Finally, the sexual hormones, with androgens and estrogens, are fundamental for the formation of the sexual characteristics and the estrous cycle. Biosynthesis of all steroid hormones (Fig. 1) is initiated by CYP11A1 with the side chain cleavage of cholesterol yielding pregnenolone [1], [2]. This rate-limiting step of the steroid hormone biosynthesis is carried out in the inner mitochondrial membrane and displays three hydroxylation reactions. In the first step, carbon C22 of cholesterol is hydroxylated followed by an oxidative attack at C20, forming the intermediate 20,22-dihdroxycholesterol. The cleavage of the side chain of cholesterol is initiated by a third hydroxylation. The electrons necessary oxygen activation and substrate hydroxylation are transferred from NAD(P)H via a NAD(P)H-depending ferredoxin reductase, adrenodoxin reductase, and a ferredoxin, adrenodoxin [3]. The product pregnenolone serves as precursor for mineralocorticoids, glucocorticoids, as well as DHEA and its derived sexual hormones.

To induce their biological activities, according to recent hypothesis steroid hormones have to be available in an unconjugated form to interact with their corresponding receptors. Conjugated steroids with sulfate groups have been regarded for a long time to be exclusively designated for excretion [4], although many mammalian species produce, predominantly during pregnancy, huge amounts of sulfonated steroids [5]–[7]. Besides their function as an inactive reservoir for steroid hormones [8], their physiological role still needs to be elucidated. So far cholesterol sulfate (CS) represents the only sulfonated steroid, which is rather well investigated: as a compound of cell membranes CS possesses a stabilizing role, CS is involved in regulating the activity of serine proteases and it is important for keratinocyte differentiation [9]. Moreover, Tuckey could demonstrate the ability of CYP11A1 to metabolize CS yielding pregnenolone sulfate [10], which can enter the “sulfatase pathway” or be metabolized to further sulfonated steroids [11]. In addition, CS was shown to have an inhibitory effect on the CYP11A1 dependent side chain cleavage of cholesterol [12], [13]. These findings show the direct involvement of sulfonated steroids in the steroid hormone biosynthesis and since the discovery of the co-localization of estrogen receptors, steroid sulfatases and estrogen sulfotransferases in the same tissue [14], new hypotheses on the role of sulfonated steroids and a system that controls the availability of free steroids arose. However, detailed investigations about the interplay between steroid biosynthesis and steroid conjugation are missing so far.

In contrast to unconjugated steroids, sulfonated steroids need to be actively transported through cell membranes. Sodium dependent organic anion transporters (SOAT) were found to possess a high sensitivity towards sulfonated steroids [15], although it has not been investigated so far whether these transporters are localized in mitochondrial membranes allowing sulfonated steroids to enter. On the other hand, sulfonated cholesterol has been found to be an endogenous compound of mitochondria in rats [16] and to be converted by CYP11A1 in isolated mitochondria [10]. This suggests that sulfonated steroids are able to pass the mitochondrial membranes and thus have direct contact with CYP11A1.

We focused here on the impact of sulfonated DHEA on CYP11A1 activity, as this steroid is the predominant circulating one in humans [17], [18]. The naturally occurring concentrations of DHEAS and DHEA have been determined to be around 10 µM and 10 nM, respectively, in young adults [19]. Moreover, the DHEAS level is increased in some adrenocortical disorders: people suffering from Cushing's syndrome due to adrenal hyperplasia display very high levels of serum DHEAS [20]. Furthermore, in Western countries, dietary supplements reach a major significance in nutrition, especially when anti-aging effects are ascribed to them. One very popular dietary supplement is DHEA or DHEAS, to which multiple beneficial implications, such as anti-diabetic effect, prevention of osteoporosis, anti-obesity action, prevention of atherosclerosis, anti-carcinogenic action and anti-aging effects are attributed [21]. Normally, 50 mg per day are administrated [22], but also up to 200 mg per day were reported to be taken [23]. However, the direct action of DHEAS on the steroid hormone biosynthesis has not been studied so far and remains an enigma.

Therefore, in this study, the influence of DHEAS on reactions catalyzed by CYP11A1 is investigated on a molecular level in a reconstituted *in vitro* system. It could be demonstrated that DHEAS but not DHEA increases the CYP11A1-dependent pregnenolone formation caused by improved cholesterol and redox partner binding.

## Experimental Procedure

### Protein expression and purification

Heterologous expression of bovine adrenodoxin reductase (AdR) and bovine adrenodoxin (Adx) was performed and the proteins were purified as previously described [24], [25]. Recombinant CYP11A1 was expressed in *E. coli* C43(DE3) cells, which were co- transformed with a plasmid encoding for groEL/groES chaperones [26].

The subsequent CYP11A1 expression was performed as described elsewhere [27]. The obtained cell pellet was suspended in 5 ml/g wet mass buffer, consisting of 50 mM potassium phosphate, pH 7.4, 500 mM sodium acetate, 20% glycerol, 1.5% sodium cholate, 1.5% Tween20, 0.1 mM EDTA, 0.1 mM DTT, 0.1 mM PMSF and 10 µM cholesterol. The disruption of the cells was carried out by sonication for 15 min with 15 s intervals, utilizing Bandelin Sonoplus with the sonotrode TT13 (Bandelin electronic, Berlin; Germany) at an amplitude of 15 Hz in an ice water bath under uninterrupted stirring. To remove cell derbis and unsoluble proteins, the homogenate was centrifuged at 30000 rpm (Himac CP75b, rotor P45, Hitachi, Tokio; Japan) for 30 min at 4°C. The supernatant was applied to an IMAC Ni-NTA column, equilibrated with buffer A, containing 50 mM potassium phosphate, pH 7.4, 500 mM sodium acetate, 20% glycerol, 1% sodium cholate, 1% Tween 20, 0.1 mM EDTA, 0.1 mM DTT, 0.1 mM PMSF and 10 µM cholesterol. Unspecific binding of proteins were removed using a washing buffer similar to buffer A, but with additional 40 mM imidazole. Elution of CYP11A1 was performed with buffer A and 200 mM imidazole. As the presence of imidazole in buffers changes the pH, acetic acid was used for readjustment to pH 7.4. Afterwards, the eluate was concentrated to a final volume of 5 ml by centricons with a size exclusion of 30 kDa. The eluate was diluted 1∶3 with buffer B (20% glycerol, 0.1 mM EDTA, 0.1 mM DTT and 10 µM cholesterol) and loaded onto an ion exchange SP- sepharose column, which was equilibrated with buffer C (20 mM potassium phosphate, pH 7.4, 20% glycerol, 10 mM imidazole, 0.1 mM EDTA, 0.1 mM DTT, 0.1 mM PMSF and 10 µM cholesterol). Weakly bound proteins were removed with a washing step using buffer C. Then CYP11A1 was eluted with 0–150 mM NaCl gradient in buffer C. The red fractions containing CYP11A1 were concentrated and diluted several times utilizing a centrifugal device to substitute the buffer C with buffer D (50 mM potassium phosphate, pH 7.4, 20% glycerol, 0.1 mM EDTA, 0.1 mM dithiothreitol, 1% sodiumcholate, 0.05% Tween 20).

### UV/Vis spectroscopy

The protein concentration of the redox partners were determined using ε_450_ = 11.3 (mM cm)^−1^ for AdR and ε_414_ = 9.8 (mM cm)^−1^ for Adx [28], [29]. CYP11A1 concentration was defined, performing a reduced carbon-monoxide difference spectroscopy according to Omura and Sato [30] with ε_448_ = 91 (mM cm)^−1^. Binding of cholesterol was investigated using difference spectroscopy, which was carried out in tandem cuvettes according to Schenkman [31]. The tandem cuvettes consist of two chambers. The first chamber of the cuvette was filled with buffer and 2 µM of CYP11A1, whereas to the second chamber only buffer was added. For reference, a second tandem cuvette was used, which was filled equally. To the first chamber of the cuvette increasing amounts of cholesterol were added with or without 5-fold excess of DHEAS. The same amount of these compounds was added to the second chamber of the reference cuvette. Cholesterol, as well as DHEAS, were dissolved in DMSO. The buffer utilized was composed of 50 mM potassium phosphate (pH 7.4), 20% glycerol, 0.5% sodium cholate and 0.05% Tween 20. Difference spectra were recorded from 370 to 450 nm. To determine the dissociation constant (*K*_d_), the values from five titrations were averaged and the resulting plots were fitted with hyperbolic regression.

### Enzyme activity assay

*In vitro* substrate conversion assays were performed as described elsewhere [32] with slight modifications. The conversion buffer (50 mM HEPES, pH 7.4, 0.05% Tween20) contained 1 μM CYP11A1, 0.5 μM AdR, 20 μM Adx, 1 mM MgCl_2_, 5 mM glucose-6-phosphate, 1 U glucose-6-phosphate dehydrogenase and 15 μM substrate or 15 µM substrate and 75 µM DHEA(S). After starting the reaction with 1 mM NADPH at 37°C for 7 min, the conversion was stopped in a boiling water bath for 5 min. Cholesterol and the resulting product pregnenolone had to be converted to the corresponding 3-one-4-en form by cholesterol oxidase, to be detectable at 240 nm during HPLC analysis. Therefore, cholesterol oxidase was added to the boiled reaction mixture and incubated for 40 min at 37°C according to Yamato [33]. Cortisol was added as internal standard. The reaction was stopped by adding 1 reaction volume ethylacetate. Extraction of steroids was performed twice with ethylacetate and the ethylacetate phase was evaporated. The steroids were resuspended in 20% acetonitrile for subsequent HPLC analysis. K_m_ and k_cat_ values were determined by plotting the substrate conversion velocities versus the corresponding substrate concentrations and by using Michaelis–Menten kinetics (hyperbolic fit) utilizing the program OriginPro 8.6G.

### HPLC analysis

Steroids were separated on a Jasco reversed phase HPLC system LC2000 using a 4.6 mm×125 mm NucleoDur C18 Isis Reversed Phase column (Macherey-Nagel) with an acetonitril/water gradient and a flow rate gradient of 1 ml/min –2 ml/min. Detection of the steroids was performed at 240 nm within 25 min at 40°C.

### Optical biosensor measurements

The interaction of Adx and CYP11A1, was assayed with a Biacore3000 system as described previously [27]. The formation of the CYP11A1-Adx complex was analyzed under three different conditions: in absence of cholesterol, in the presence of cholesterol (CYP11A1concentration: cholesterol concentration, 1∶10) and in the presence of cholesterol and DHEAS (CYP11A1 concentration: cholesterol concentration: DHEAS concentration, 1∶10∶50). CYP11A1 was injected in concentrations between 5 nM and 7.5 nM in HBS-EP buffer at least three times for each concentration. Removal of bound CYP11A1 utilizing 2 mM NaOH was done as described elsewhere [34]. Biaeval 4.1 software was used for determination of *K*_d_ values.

*Statistical evaluation*- To determine the distribution of the samples Kolmogorov-Smirnov-test was used. To determine statistically significant differences between control samples and samples incubated with DHEA or DHEAS, Mann-Whitney *U*-test was applied. One asterisk symbolizes p<0.05, and two asterisks p<0.01.

## Results

### Optimization of the expression of CYP11A1 in *E. coli*

**Figure 2:**
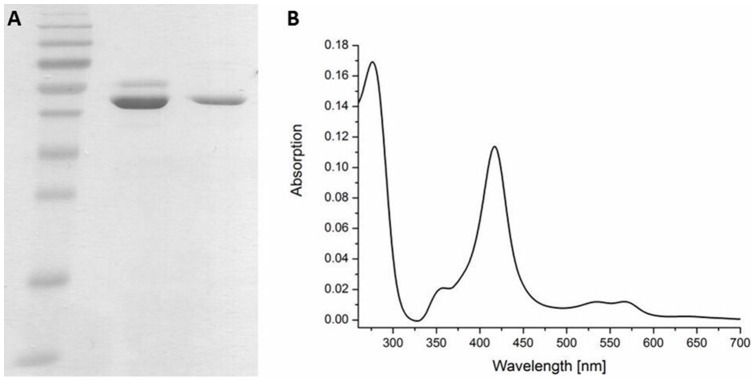
A) SDS-Polyacrylamid gel electrophoresis. Lane 1: protein marker IV (peqlab), lane 2: CYP11A1 after Ni-NTA purification, lane 3: CYP11A1 after Ni-NTA and Sp-sepharose purification. B) Absolute spectrum of purified CYP11A1 in the range of 260–700 nm.

The heterologous expression of CYP11A1 in *E coli* has been reported to be in the range between 45–55 nmol l^−1^ culture [27], [36], which is rather low. Harnastai et al. were able to enhance recombinant CYP11A1 expression up to 430 nmol l^−1^ culture co-expressing HemA [37]. In order to further improve the expression level of CYP11A1 we utilized the C43DE3 *E. coli* strain as expression host, which was shown to be adequate for other membrane-bound P450s, as previous results demonstrated [32], [34], [35]. The expressed His-tagged protein was purified by IMAC Ni-NTA followed by an ion exchange SP-sepharose. The purity of the enzyme was determined via SDS gelelectrophoresis (Fig. 2a), in which a single band at 55 kDa was obtained. The enzyme shows a typical P450 CO-difference spectrum with a major peak at 450 nm and a minor one at 420 nm (not shown). The expression levels using *E. coli* strain C43DE3 achieved here were 640 nmol per liter culture after the two purification steps, which is a great improvement compared to the published results. The UV/Vis spectrum classifies the expressed CYP11A1 as a low-spin protein with the typical cytochrome P450 absorption spectrum: the Q bands at 569 nm (α-band) and 541 nm (β-band), the Soret band at 418 nm, the UV band at 360 nm and the protein-band at 278 nm (Fig. 2b).

### CYP11A1 dependent pregnenolone formation in the presence of sulfonated DHEA

CYP11A1 catalyzes the first reaction step in human steroidogenesis, the conversion of cholesterol to pregnenolone with a three step hydroxylation preceding the side-chain cleavage between C20 and C22. In this study, we investigated the influence of sulfonated DHEA on the CYP11A1 catalyzed reaction on a molecular level.

**Figure 3:**
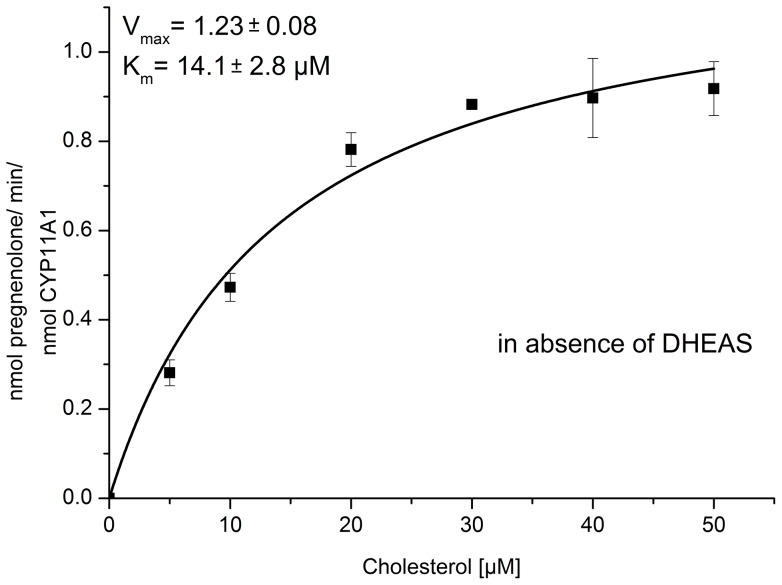
Kinetics of CYP11A1. Determination of kinetic parameters for the conversion of cholesterol to pregnenolone catalyzed by CYP11A1 using Adx and AdR as redox partners. The product formation analyzed by HPLC is represented as mean ± standard deviation of four individual experiments.

For detection of the product at 240 nm, pregnenolone was transformed to progesterone by using cholesterol oxidase reaction. The kinetic parameters of pregnenolone formation were determined to be K_m_ = 14.1±2.8 µM and k_cat_ = 1.23±0.08 min^−1^ (Fig. 3). In a first series of experiments, it was checked whether DHEAS can bind to CYP11A1 causing spectral changes of the protein. It was shown that DHEAS up to concentrations of 500 µM did not cause any spectral changes excluding its binding as type I or type II substrate (data not shown).

**Figure 4:**
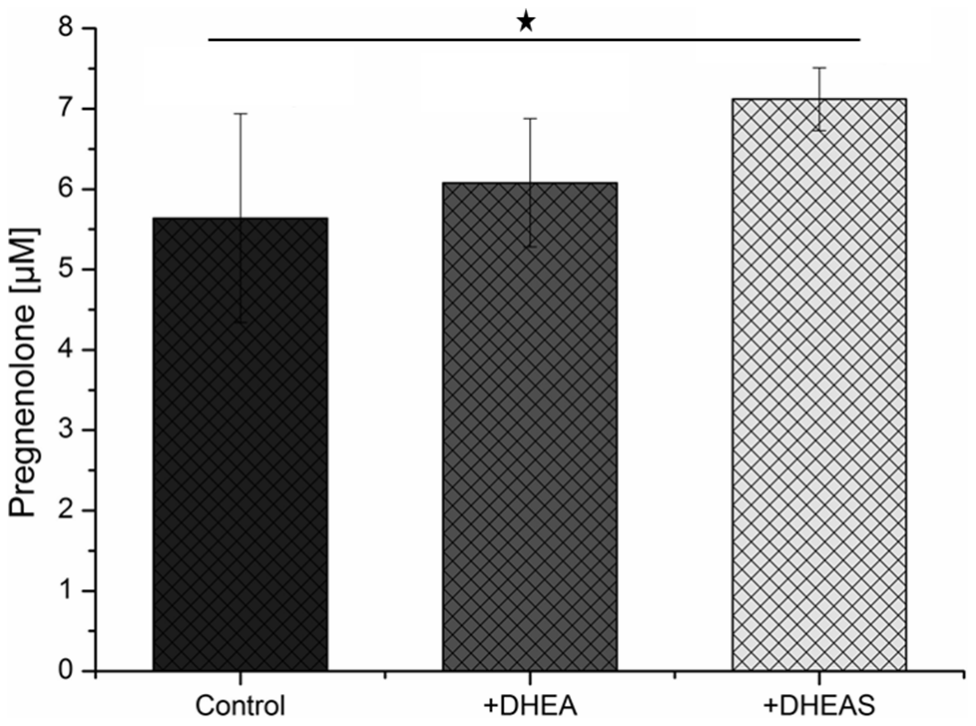
Comparison of pregnenolone formation by CYP 11A1 in the presence of 5-fold excess of DHEA or DHEAS (n = 5 for each group, asterisk: p<0.05).

For further investigation of the DHEAS induced effect on the first step in steroidogenesis, we studied the activity of CYP11A1-dependent cholesterol side chain cleavage in the presence of 75 µM DHEA or DHEAS at a cholesterol concentration of 15 µM, which is around the K_m_ value determined (Fig. 3). The evaluation of the HPLC data is shown in Figure 4. The amount of pregnenolone formed was 5.6±1.3 µM for the control experiment without DHEAS or DHEA and 6±0.8 µM for the sample incubated with a 5-fold excess of DHEA. In contrast, the CYP11A1 dependent activity in the presence of DHEAS significantly increased by 26% forming 7.1±0.4 µM pregnenolone.

**Figure 5:**
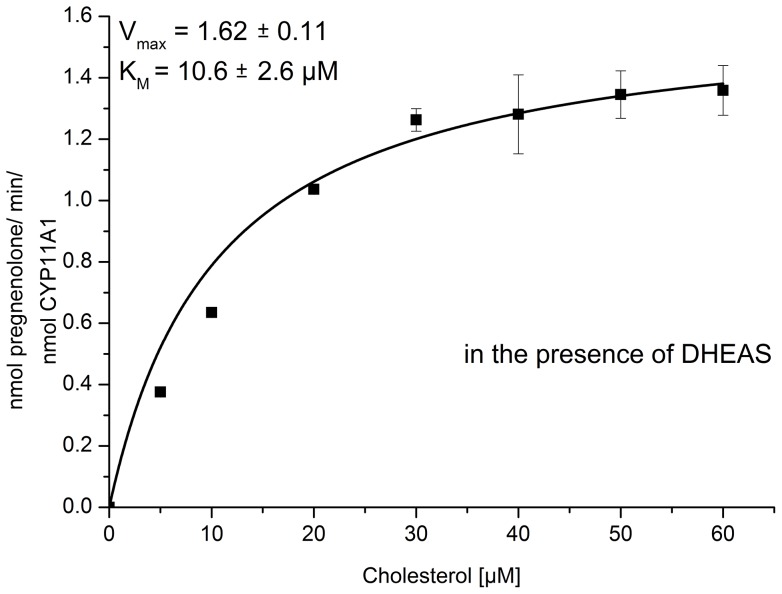
Kinetics of CYP11A1. Determination of kinetic parameters for the conversion of cholesterol to pregnenolone catalyzed by CYP11A1 with Adx and AdR as redox partners in the presence of DHEAS. The product formation analyzed by HPLC is represented as mean ± standard deviation of four individual experiments.

After observing an increased product formation of the CYP11A1 catalyzed reaction in the presence of DHEAS, it was interesting to investigate the influence of DHEAS on the kinetics of this reaction. As shown in Figure 5 and Table 1, in the presence of DHEAS the K_m_ value for cholesterol decreased while the k_cat_ value increased being 10.6±2.6 µM and 1.62±0.1 min^−1^, respectively. This leads to a nearly two fold increase of the catalytic efficiency of CYP11A1 in the presence of DHEAS being 87 (min^−1^mM^−1^) in absence and 153 (min^−1^mM^−1^) in the presence of DHEAS.

**Table 1:** Kinetic parameters of CYP11A1, metabolizing cholesterol with or without the addition of DHEAS.

− DHEAS			+ DHEAS		
K_m_ (mM)	k_cat_ (min^−1^)	k_cat/_K_m_ (min^−1^mM^−1^)	K_m_ (mM)	k_cat_ (min^−1^)	k_cat/_K_m_ (min^−1^mM^−1^)
0.0142±0.0028	1.23±0.08	86.6	0.0106±0.0026	1.62±0.11	152.8

### Analysis of the molecular mechanism of DHEAS action on CYP11A1

**Figure 6:**
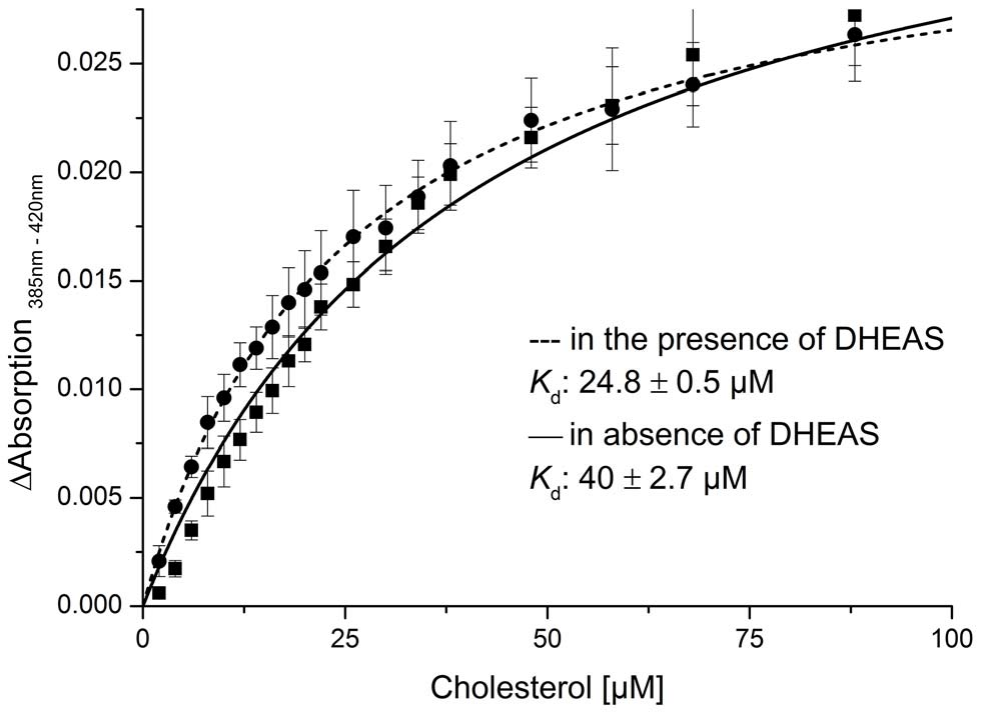
Determination of substrate affinity to CYP11A1. *K*_d_ values of CYP11A1 for cholesterol in absence (n = 5) and in the presence of a 5-fold excess of DHEAS (n = 5).

To investigate in more detail, which reaction step is being affected by DHEAS, we studied the binding of the substrate and the redox partner, Adx, in absence and in the presence of DHEAS. As shown in Figure 6, the *K*_d_ value of CYP11A1 and cholesterol is 40±2.7 µM in absence and 24.8±0.5 µM in the presence of DHEAS.

This indicates that the affinity of cholesterol for CYP11A1 is increased about 2-fold in the presence of DHEAS.

**Figure 7:**
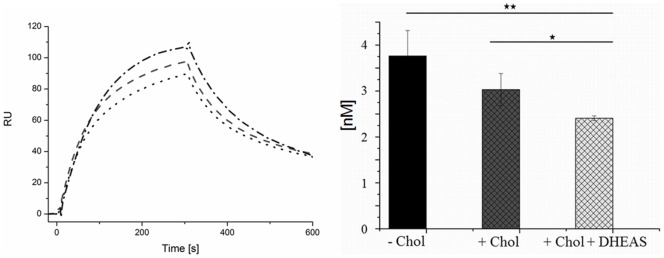
Binding affinity of Adx and CYP11A1. a) Overlay of Biacore3000 sensograms of measurements with 7.5 nM CYP11A1 in absence of cholesterol, in the presence of cholesterol and in the presence of cholesterol and DHEAS (CYP11A1: cholesterol: DHEAS, 1∶15∶75), b) Comparison of *K*_d_ values for the interaction between CYP11A1 and Adx without cholesterol (n = 8), with cholesterol (n = 8, CYP11A1: cholesterol, 1∶15) and with cholesterol in the presence of DHEAS (n = 8, CYP11A1: cholesterol: DHEAS, 1∶15∶75).

For the determination of the dissociation constant of CYP11A1 and Adx, surface plasmon resonance measurements were conducted (Fig. 7). Binding of CYP11A1 and Adx is essential for the correct function of the side-chain cleavage, since 6 electrons have to be transported from NADPH via AdR and Adx to CYP11A1. Surface plasmon resonance measurements using CYP11A1 and Adx revealed *K*_d_ values of 3.8±0.55 nM, when the interaction was measured without substrate, of 3.0±0.35 nM with addition of cholesterol and of 2.4±0.05 nM in the presence of cholesterol and DHEAS (Fig. 7). Comparing the interaction of CYP11A1 and Adx in the presence of cholesterol and DHEAS with the interaction of CYP11A1 and Adx in the presence of only cholesterol, a decrease of the *K*_d_ value by 20% is achieved, while a decrease of the *K*_d_ by 36% can be observed when the *K*_d_ values without substrate and with substrate and DHEAS are compared. As demonstrated in Table 2, changes in the interaction between CYP11A1 and Adx by DHEAS are mainly caused by changes in the *k*_on_ values. In the presence of cholesterol DHEAS increases the *k*_on_ value for the binding of Adx to CYP11A1 by 48%, whereas the *k*_on_ value is increased by 110% compared with the sample in absence of cholesterol. Both results display statistical significance with p<0.01. The *k*_off_ values do not differ in a statistically significant manner.

**Table 2:** Binding parameters for CYP11A1 and Adx without cholesterol (n = 8), with cholesterol (n = 8) and with cholesterol and in the presence of DHEAS (n = 8) determined from measurements with a Biacore3000 system.

− Chol		+ Chol		+Chol +DHEAS	
*k*_on_ (M^−1^ s^−1^)	*k*_off_ (s^−1^)	*k*_on_ (M^−1^ s^−1^)	*k*_off_ (s^−1^)	*k*_on_ (M^−1^ s^−1^)	*k*_off_ (s^−1^)
6.4±1.84	2.4±0.99	8.9±0.79	2.8±0.09	13.2±2.29	3.0±0.14

## Discussion

In human organism, 99% of DHEA circulates in its sulfonated form, DHEAS [38]. The concentration of circulating DHEAS reaches up to 10 µM in young adults [19] and decreases afterwards with age with concentrations of DHEAS between 20–30% in 70–80 year old people compared with the value of young adults [39]. These characteristics are unique for DHEA or DHEAS and in contrast to other steroid hormones like aldosterone or cortisol, whose serum concentrations stay constant during life. Hence, DHEAS is regarded as “fountain of youth”, leading to uncontrolled use in Western countries [38]. In the last decades a huge number of publications concerning the effect of DHEAS on health appeared and the results were really conflicting, reporting about insignificant activity and even harmful effects up to life extending properties of DHEA or DHEAS. Long-term studies about the effect of DHEAS on health are very scarce and the existing ones concern elderly people, where the intake of DHEA is limited to amounts necessary to establish DHEAS concentrations that people have in younger age. This DHEAS replacement therapy in elderly people seems to have beneficial effects concerning bone turnover, skin hydration and libido, increasing the well-being of aging persons [22]. However, since DHEAS reaches major significance as dietary supplement, this requires further research on the effect of high DHEAS concentrations on metabolism. So far, research was mainly focused on the effect of DHEA and DHEAS on the production of androgens and estrogens. The effect of DHEA or DHEAS on the whole steroid hormone biosynthesis, especially on the enzymes involved in the first steps is not investigated so far. The rate-limiting step of the steroid hormone biosynthesis is realized by CYP11A1 catalyzing the side chain cleavage of cholesterol to yield pregnenolone. Thus, CYP11A1 represents an adequate target for regulation, on which the effect of DHEAS was studied here in a reconstituted *in vitro* system. In the presence of DHEAS, CYP11A1 displayed a 26% increased pregnenolone formation compared to the control, while in the presence of DHEA no significant difference from the control could be observed. Thus, the negatively charged sulfate group of DHEAS seems to be essential for the increase of the CYP11A1 activity. Interestingly, it was previously shown that polycationic natural polyamines interact through electrostatic forces with negatively charged spots at protein interfaces of the CYP11A1-Adx system. This interaction of the positively charged polyamines with the CYP11A1-Adx interface leads to an inhibition of the CYP11A1 activity [40]. This indicates that the type of charge of the compounds (DHEAS or polyamines) determines whether it displays an activatory (DHEAS) or inhibitory (polyamines) effect on the CYP11A1-dependent reaction, with an anionic compound stimulating and a cationic compound inhibiting CYP11A1 activity. To examine the stimulatory effect of DHEAS at the molecular level, we investigated the individual steps of the reaction cycle. It was demonstrated that the binding of the CYP11A1 substrate cholesterol was improved (Fig. 5) by the presence of DHEAS. Likewise, the interaction with the redox partner, Adx, was promoted by DHEAS. Together with the observation that DHEAS increases the catalytic efficiency of CYP11A1 nearly two-fold, this indicates that improved substrate and Adx binding to CYP11A1 cause higher activity and product formation.

Interestingly, the dissociation of the CYP11A1-Adx complex was not influenced when DHEAS was added similar to the situation with polyamines [40], as the similar *k*_off_ values between the samples incubated with only cholesterol and the samples incubated with cholesterol in the presence of DHEAS showed. This demonstrates that the interaction between CYP11A1 and Adx is facilitated as the *k*_on_ values display, probably through slight conformational changes of CYP11A1 and/or Adx induced by the presence of DHEAS.

As the crystal structures of CYP11A1 and of the CYP11A1-Adx complex are available, docking studies with DHEAS were performed to get a deeper insight concerning the structure- and function- relationships (data not shown). In fact, several amino groups of the CYP11A1-Adx complex seem to be able to establish contacts to DHEAS due to its negatively charged sulfate group, leading to various putative binding sites for DHEAS. This fact makes it impossible to postulate a certain binding position of DHEAS which could explain the obtained results by a reasonable way without further experiments.

Whether the stimulatory effect of DHEAS on the CYP11A1-system might possess physiological meaning and displays a novel putative regulation of the steroid hormone biosynthesis on the cellular level, needs to be investigated. It has to be mentioned, that the concentration of DHEAS used in this work (75 µM) exceeds the plasma concentration in humans (10 µM). However, it is not known whether DHEAS is enriched in tissues. In addition, elevated DHEAS concentrations up to 10-fold might occur due to adrenocortical disorders or the additional dietary intake of DHEA or DHEAS and do not only increase the level of steroids designated for the production of sexual hormones, but also might affect the steroid biosynthesis through an increased production of pregnenolone, which represents the precursor for mineralocorticoids, glucocorticoids and sexual hormones. As a consequence, the level of the end products of the steroid hormone biosynthesis might also increase, which could cause unwanted effects on health. Elevated production of the mineralocorticoid aldosterone leads to increasing blood pressure while an increase in the production of the glucocorticoid cortisol leads to Cushing's syndrome. Thus, besides all the beneficial effects ascribed to DHEAS, intake of high amounts of DHEAS might cause undesired side effects.

Taken together, our results clearly demonstrate that the catalytic efficiency of the side-chain cleavage of cholesterol can be up-regulated by 75% in the presence of DHEAS but not DHEA. The molecular mechanism of this increase was shown to be due to improved binding of the substrate cholesterol and the redox partner, Adx.

## References

[1] Lisurek M, Bernhardt R (2004) Modulation of aldosterone and cortisol synthesis on the molecular level. Molecular and cellular endocrinology 215, 149–159. 10.1016/j.mce.2003.11.008 15026188

[2] Bernhardt R, Waterman MR (2007) Cytochrome P450 and Steroid Hormone Biosynthesis. The Ubiquitous Roles of Cytochrome P450 Proteins, John Wiley & Sons, Ltd. 361–396.

[3] Hannemann F, Bichet A, Ewen KM, Bernhardt R (2007) Cytochrome P450 systems – biological variations of electron transport chains. Biochim Biophys Acta. 1770, 330–344. Epub 2006 Aug 2002. 10.1016/j.bbagen.2006.07.017 16978787

[4] Strott CA (1996) Steroid sulfotransferases. Endocrine reviews 17, 670–697. 10.1210/edrv-17-6-670 8969973

[5] Janowski T, Zdunczyk S, Ras A, Mwaanga ES (1999) Use of estrone sulfate determination in goat blood for the detection of pregnancy and prediction of fetal number. Tierarztliche Praxis. Ausgabe G, Grosstiere/Nutztiere 27, 107–109. 10326236

[6] Claus R, Hoffmann B (1980) Oestrogens, compared to other steroids of testicular origin, in blood plasma of boars. Acta Endocrinol (Copenh) 94, 404–411. 10.1530/acta.0.0940404 7191612

[7] Hoffmann B, Landeck A (1999) Testicular endocrine function, seasonality and semen quality of the stallion. Anim Reprod Sci. 57, 89–98. 10.1016/s0378-4320(99)00050-0 10565441

[8] Pasqualini JR, Chetrite GS (2005) Recent insight on the control of enzymes involved in estrogen formation and transformation in human breast cancer. J Steroid Biochem Mol Biol. 93, 221–236. 10.1016/j.jsbmb.2005.02.007 15860265

[9] Strott CA, Higashi Y (2003) Cholesterol sulfate in human physiology: what's it all about? J Lipid Res. 44, 1268–1278. 10.1194/jlr.R300005-JLR200 12730293

[10] Tuckey RC (1990) Side-chain cleavage of cholesterol sulfate by ovarian mitochondria. The Journal of steroid biochemistry and molecular biology 37, 121–127. 10.1016/0960-0760(90)90380-4 2242345

[11] Korte K, Hemsell PG, Mason JI (1982) Sterol sulfate metabolism in the adrenals of the human fetus, anencephalic newborn, and adult. The Journal of clinical endocrinology and metabolism 55, 671–675. 10.1210/jcem-55-4-671 6213632

[12] Lambeth JD, Xu XX, Glover M (1987) Cholesterol sulfate inhibits adrenal mitochondrial cholesterol side chain cleavage at a site distinct from cytochrome P-450scc. Evidence for an intramitochondrial cholesterol translocator. The Journal of biological chemistry 262, 9181–9188. 3597410

[13] Tsutsumi R, Hiroi H, Momoeda M, Hosokawa Y, Nakazawa F, et al.. (2008) Inhibitory effects of cholesterol sulfate on progesterone production in human granulosa-like tumor cell line, KGN. Endocrine journal 55, 575–581. 10.1507/endocrj.k07-097 18490834

[14] Schuler G, Greven H, Kowalewski MP, Doring B, Ozalp GR, et al.. (2008) Placental steroids in cattle: hormones, placental growth factors or by-products of trophoblast giant cell differentiation? Exp Clin Endocrinol Diabetes. 116, 429–436. 10.1055/s-2008-1042408 18704836

[15] Geyer J, Godoy JR, Petzinger E (2004) Identification of a sodium-dependent organic anion transporter from rat adrenal gland. Biochem Biophys Res Commun. 316, 300–306. 10.1016/j.bbrc.2004.02.048 15020217

[16] Xu XX, Lambeth JD (1989) Cholesterol sulfate is a naturally occurring inhibitor of steroidogenesis in isolated rat adrenal mitochondria. The Journal of biological chemistry 264, 7222–7227. 2708364

[17] Montanini V, Simoni M, Chiossi G, Baraghini GF, Velardo A, et al.. (1988) Age-related changes in plasma dehydroepiandrosterone sulphate, cortisol, testosterone and free testosterone circadian rhythms in adult men. Hormone research 29, 1–6. 10.1159/000180956 2969362

[18] Nieschlag E, Loriaux DL, Ruder HJ, Zucker IR, Kirschner MA, et al.. (1973) The secretion of dehydroepiandrosterone and dehydroepiandrosterone sulphate in man. The Journal of endocrinology 57, 123–134. 10.1677/joe.0.0570123 4349596

[19] Baulieu EE (1996) Dehydroepiandrosterone (DHEA): a fountain of youth? The Journal of clinical endocrinology and metabolism 81, 3147–3151. 10.1210/jcem.81.9.8784058 8784058

[20] Sekihara H, Osawa N, Ibayashi H (1972) A radioimmunoassay for serum dehydroepiandrosterone sulfate. Steroids 20, 813–824. 10.1016/0039-128x(72)90059-1 4265707

[21] Nawata H, Yanase T, Goto K, Okabe T, Ashida K (2002) Mechanism of action of anti-aging DHEA-S and the replacement of DHEA-S. Mechanisms of ageing and development 123, 1101–1106. 10.1016/s0047-6374(01)00393-1 12044959

[22] Baulieu EE, Thomas G, Legrain S, Lahlou N, Roger M, et al.. (2000) Dehydroepiandrosterone (DHEA), DHEA sulfate, and aging: contribution of the DHEAge Study to a sociobiomedical issue. Proceedings of the National Academy of Sciences of the United States of America 97, 4279–4284. 10.1073/pnas.97.8.4279 PMC18228 10760294

[23] Vacheron-Trystram MN, Cheref S, Gauillard J, Plas J (2002) A case report of mania precipitated by use of DHEA. Encephale. 28, 563–566. 12506269

[24] Uhlmann H, Beckert V, Schwarz D, Bernhardt R (1992) Expression of bovine adrenodoxin in E. coli and site-directed mutagenesis of/2 Fe-2S/cluster ligands. Biochemical and biophysical research communications 188, 1131–1138. 10.1016/0006-291x(92)91349-u 1332711

[25] Sagara Y, Barnes HJ, Waterman MR (1993) Expression in Escherichia coli of functional cytochrome P450c17 lacking its hydrophobic amino-terminal signal anchor. Archives of biochemistry and biophysics 304, 272–278. 10.1006/abbi.1993.1349 8323292

[26] Nishihara K, Kanemori M, Kitagawa M, Yanagi H, Yura T (1998) Chaperone coexpression plasmids: differential and synergistic roles of DnaK-DnaJ-GrpE and GroEL-GroES in assisting folding of an allergen of Japanese cedar pollen, Cryj2, in Escherichia coli. Applied and environmental microbiology 64, 1694–1699. 10.1128/aem.64.5.1694-1699.1998 PMC106217 9572938

[27] Janocha S, Bichet A, Zollner A, Bernhardt R (2011) Substitution of lysine with glutamic acid at position 193 in bovine CYP11A1 significantly affects protein oligomerization and solubility but not enzymatic activity. Biochimica et biophysica acta 1814, 126–131. 10.1016/j.bbapap.2010.06.002 20538078

[28] Kimura T, Ono H (1968) Preparation of testis non-heme iron protein and substitution for adrenodoxin by various non-heme iron proteins in steroid 11-beta-hydroxylation. Journal of biochemistry 63, 716–724. 10.1093/oxfordjournals.jbchem.a128836 4387149

[29] Hiwatashi A, Ichikawa Y, Maruya N, Yamano T, Aki K (1976) Properties of crystalline reduced nicotinamide adenine dinucleotide phosphate-adrenodoxin reductase from bovine adrenocortical mitochonria. I. Physicochemical properties of holo- and apo-NADPH-adrenodoxin reductase and interaction between non-heme iron proteins and the reductase. Biochemistry 15, 3082–3090. 10.1021/bi00659a023 986153

[30] Omura T, Sato R (1964) The Carbon Monoxide-Binding Pigment of Liver Microsomes. Ii. Solubilization, Purification, and Properties. The Journal of biological chemistry 239, 2379–2385. 14209972

[31] Schenkman JB (1970) Studies on the nature of the type I and type II spectral changes in liver microsomes. Biochemistry 9, 2081–2091. 10.1021/bi00812a009 4245596

[32] Hobler A, Kagawa N, Hutter MC, Hartmann MF, Wudy SA, et al.. (2012) Human aldosterone synthase: recombinant expression in E. coli and purification enables a detailed biochemical analysis of the protein on the molecular level. The Journal of steroid biochemistry and molecular biology 132, 57–65. 10.1016/j.jsbmb.2012.03.002 22446688

[33] Yamato S, Nakagawa S, Yamazaki N, Aketo T, Tachikawa E (2010) Simultaneous determination of pregnenolone and 17alpha-hydroxypregnenolone by semi-micro high-performance liquid chromatography with an immobilized cholesterol oxidase as a pre-column reactor: application to bovine adrenal fasciculata cells. Journal of chromatography. B, Analytical technologies in the biomedical and life sciences 878, 3358–3362. 10.1016/j.jchromb.2010.10.020 21081289

[34] Zollner A, Kagawa N, Waterman MR, Nonaka Y, Takio K, et al.. (2008) Purification and functional characterization of human 11beta hydroxylase expressed in Escherichia coli. The FEBS journal 275, 799–810. 10.1111/j.1742-4658.2008.06253.x 18215163

[35] Kagawa N (2011) Efficient expression of human aromatase (CYP19) in E. coli. Methods Mol Biol. 705, 109–122. 10.1007/978-1-61737-967-3_7 21125383

[36] Wada A, Mathew PA, Barnes HJ, Sanders D, Estabrook RW, et al.. (1991) Expression of functional bovine cholesterol side chain cleavage cytochrome P450 (P450scc) in Escherichia coli. Archives of biochemistry and biophysics 290, 376–380. 10.1016/0003-9861(91)90554-v 1929405

[37] Harnastai IN, Gilep AA, Usanov SA (2006) The development of an efficient system for heterologous expression of cytochrome P450s in Escherichia coli using hemA gene co-expression. Protein expression and purification 46, 47–55. 10.1016/j.pep.2005.07.006 16122943

[38] Leowattana W (2004) DHEAS as a new diagnostic tool. Clinica chimica acta; international journal of clinical chemistry 341, 1–15. 10.1016/j.cccn.2003.10.031 14967152

[39] Kroboth PD, Salek FS, Pittenger AL, Fabian TJ, Frye RF (1999) DHEA and DHEA-S: a review. Journal of clinical pharmacology 39, 327–348. 10.1177/00912709922007903 10197292

[40] Berwanger A, Eyrisch S, Schuster I, Helms V, Bernhardt R (2010) Polyamines: naturally occurring small molecule modulators of electrostatic protein-protein interactions. Journal of inorganic biochemistry 104, 118–125. 10.1016/j.jinorgbio.2009.10.007 19926138

